# Schizophrenia with obsessive-compulsive suicidal images

**DOI:** 10.1192/j.eurpsy.2022.2061

**Published:** 2022-09-01

**Authors:** V.-M. Vasadi, O. Capatina, M. Fadgyas Stanculete

**Affiliations:** 1 Cluj County Emergency Hospital, 2nd Psychiatry Department, Cluj-Napoca , Romania; 2 Iuliu Hatieganu University of Medicine and Pharmacy Cluj-Napoca , Department Of Neurosciences - Psychiatry, Cluj-Napoca , Romania

**Keywords:** schizofrenie, obsessive-compulsive, suicidal ideation, clozapine

## Abstract

**Introduction:**

Background A 19-year old patient with a history of traumatic childhood events presents herself for suicidal behavior driven by complex auditory hallucinations, self-mutilating behavior, and obsessive-compulsive suicidal images that depicted the patient lying dead on the floor with both wrists cut open. Prior to hospitalization, over the past 10 months, the patient exhibited symptoms of low mood, anhedonia, and an overall decline in school performance which led to a diagnosis of MDD with psychotic symptoms, but treatment with Duloxetine 60mg and Olanzapine 10mg proved to be inefficient.

**Objectives:**

Case Presentation Upon admission the patient’s mimic and behavior did not support the described sadness, she presented a circumstantial discourse with frequent thought blockages and delusions. Associated the patient also described hypervigilance and avoidance behavior towards men, flashbacks, vivid nightmares, and obsessive-compulsive self-mutilating impulses and images.

**Methods:**

Initially, treatment was started with Olanzapine 20mg, which was augmented two months later with Sertraline 50mg and Bromazepam 3mg. This treatment led to an incomplete resolution of the obsessive symptoms, which led to the increase of Sertraline at 100mg, but at her 1-month check-up evaluation, she presented increased suicidal ideation and daily obsessive-compulsive images.

**Results:**

The patient was lastly diagnosed with schizophrenia, and due to the persistence of the suicidal ideations and delusions and the obsessive-compulsive symptoms, the treatment plan was revised.

**Conclusions:**

Improvement of symptoms appeared under treatment with 300mg Clozapine and 50mg Fluvoxamine. In total the patient needed 98 days of hospitalization and lasting remission of symptoms appeared only under treatment with Clozapine and Fluvoxamine.

**Disclosure:**

No significant relationships.

